# Early Parenting and Infant–Parent Attachment: Developmental Origins of Psychotic Experiences

**DOI:** 10.1002/brb3.71286

**Published:** 2026-02-28

**Authors:** Andrea P. Cortes Hidalgo, Koen Bolhuis, Henning Tiemeier, Marian J. Bakermans‐Kranenburg, Marinus H. van IJzendoorn

**Affiliations:** ^1^ Department of Child and Adolescent Psychiatry/Psychology Erasmus MC Rotterdam the Netherlands; ^2^ Grupo de Investigación Clínica de la Corporación para Investigaciones Biológicas Medellín Colombia; ^3^ Department of Social and Behavioral Sciences Harvard T.H. Chan School of Public Health Boston Massachusetts USA; ^4^ William James Center for Research Ispa – Instituto Universitário Lisbon Portugal; ^5^ Department of Psychology, Personality, Social and Developmental Psychology Stockholm University Stockholm Sweden; ^6^ Facultad de Psicología y Humanidades Universidad San Sebastián Valdivia Chile; ^7^ Research Department of Clinical, Educational and Health Psychology UCL London UK; ^8^ Department of Psychiatry Monash University Melbourne Australia

**Keywords:** adversity, attachment, parenting, prospective, psychosis, psychotic symptoms

## Abstract

**Introduction:**

The infant–parent relationship is theorized to be related to the origins of psychotic experiences, given the key role of infant–parent attachment and early‐life caregiving in children's neurodevelopmental trajectories. Yet, the magnitude of this association is not well understood, and research is often based on self‐reports. We examined the relationship of disconnected and extremely insensitive parenting and disorganized infant attachment with the occurrence of psychotic experiences in childhood and adolescence. We additionally examined the role of maternal experiences of loss, a hypothesized antecedent of disconnected parenting, disorganized attachment, and psychotic experiences.

**Methods:**

This prospective study (*N* = 627) is embedded in the Generation R Study. Maternal experiences of loss within 2 years of the child's birth were self‐reported. Parenting behaviors (based on continuous scores) and the infant–parent attachment were observed when infants were 14 months old. Psychotic experiences were self‐reported via questionnaires at ages 10 and 14 years. We used a structural equation model adjusted for covariates to assess the association between maternal loss experiences, parenting behaviors, infant disorganized attachment, and psychotic experiences.

**Results:**

Extreme insensitive parenting was associated with more hallucinations and delusions at age 14 years (hallucinations OR = 1.34, 95% CI = 1.07–1.66; delusions OR = 1.31, 95% CI = 1.02–1.68). Disorganized infant attachment and disconnected parenting were not related to psychotic experiences. Maternal experiences of loss were not associated with psychotic experiences, and we found no evidence for a pathway between maternal experiences of loss, parenting behaviors, or disorganized attachment, and subsequent psychotic experiences.

**Conclusion:**

This study suggests that the role of disorganized infant–parent attachment in the risk of psychotic experiences of children from the general population might be smaller than expected. Instead, our results suggest that adverse caregiving behaviors related to harsh and maltreating parenting very early in development may predict psychotic experiences in adolescence.

## Graphical abstract

This study suggests that the role of disorganized infant–parent attachment in the risk of psychotic experiences of children from the general population might be smaller than expected. Instead, results suggest that adverse caregiving behaviors related to harsh and maltreating parenting very early in development may predict psychotic experiences in adolescence.

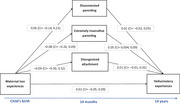

## 1 Introduction

Infant–parent relationships develop early in life and contribute to the child's neurodevelopment (Rowell and Neal‐Barnett 2022) and stress regulation system (Cicchetti and Doyle 2016; Planalp et al. 2022; van IJzendoorn et al. 1999). During infancy, children rely on their caregiver for regulating their response to stressful situations that they cannot manage on their own, and these early experiences constitute a basis for the development of their own adaptive or maladaptive regulation strategies to deal with future stress (Benoit 2004; Gunnar 2017; Tharner et al. 2012).

An “organized”, “secure” attachment relationship forms when caregivers give consistent and sensitive responses to the infant's stress, and infants see their caregiver as a secure base from which they can explore the social and physical environment as well as a safe haven to which they can come back for comfort when distressed (Madigan et al. 2024). Other types of “organized” attachment relationships include “avoidant” and “resistant,” in which, although insecure in nature because of inconsistent or rejecting caregiver responses, infants know how to respond and have an “organized” strategy to deal with stress, adapted to the limited availability of the caregiver (Benoit 2004; Groh, Narayan, et al. 2017). In contrast, a small proportion of children develop a “disorganized” attachment pattern, characterized by strikingly contradictory and incoherent behaviors when faced with stress (e.g., behavioral stereotypes, freezing, fear of the caregiver) (Bakermans‐Kranenburg et al. 2005; Groh et al. 2012; Madigan et al. 2023; van IJzendoorn et al. 1999). The parents of infants with disorganized attachment are thought to represent both the infant's source of fear and their only potential source of safety (Main and Hesse 1990; van IJzendoorn et al. 1999), and to have atypical behaviors when interacting with the infant, such as frightened facial expressions, threatening behaviors, or dissociated behavior (Schuengel et al. 1999).

Psychotic experiences (PEs) refer to the subclinical presentation of hallucinations and delusions, and are at the lower end of the psychosis spectrum, in which the top end are psychotic disorders (severe hallucinations and delusions) (Staines et al. 2022). Psychotic experiences are common in the general population, with a prevalence of 17% in childhood and around 8% in adolescence (Kelleher et al. 2012), while psychotic disorders are less common, with a lifetime prevalence of around 3% (Perälä et al. 2007). The psychosis spectrum has been hypothesized to have neurodevelopmental origins, in which early‐life (perinatal, childhood, and adolescence) factors such as an altered infant–parent attachment and dysfunctional parenting could alter the normal neurological development and result in clinical manifestations (Dolz et al. 2024; Longden and Read 2016). This is one of the reasons for which the link between disorganized infant attachment and the psychosis spectrum (Haltigan et al. 2021) has been postulated. Another reason is that disorganized attachment and neurodevelopmental problems (such as psychosis and autism spectrum disorders) have similar phenotypic characteristics, including atypical postures, behavioral stereotypes, and deficits in emotional regulation (Compton et al. 2015; Haltigan et al. 2021; Potter‐Dickey et al. 2020). Also, studies have shown that disorganized attachment is related to subsequent psychopathology, such as emotional and behavioral problems and dissociative symptoms (Sroufe 2005), and these, in turn, are related to PEs (Bolhuis, Koopman‐Verhoeff, et al. 2018). Importantly, both disorganized attachment and PEs are more common in children exposed to maltreatment (Cicchetti and Doyle 2016; Croft et al. 2019), which suggests that disorganized infant–parent attachment could be a mediator of the association between maltreatment and PEs.

A large body of literature supports the relation between childhood adverse events, such as bullying, abuse, and neglect, and the risk for PEs, and several studies have explored potential mediators of this association (Alameda et al. 2020; Sideli et al. 2020; Staines et al. 2022). A systematic review on mediators of the relation between adversity and psychosis (Sideli et al. 2020) included eight studies on the mediating role of attachment. Most of these supported a mediating role of attachment in the relation between adversity and psychosis, but all assessed current attachment styles in adulthood, and thus did not study early‐life attachment. A systematic review by Alameda and colleagues specifically examined whether attachment styles in adulthood were a potential mediator between childhood adversity and adult PEs, and found inconsistent evidence based on five studies (Alameda et al. 2020). In contrast, the evidence on disorganized infant–parent attachment as a potential mediating factor of childhood adversity and PEs is very limited. In fact, evidence on the link between infant disorganized attachment and PEs is lacking, with very few studies examining this association. For example, a case‐report study with prospective assessments of attachment described a case in which a girl with disorganized attachment was later diagnosed with childhood psychosis (Massie 1977). Apart from this case‐report study, evidence has been largely based on adolescents and adults, with a meta‐analysis reporting a relation between insecure attachment style and the psychosis spectrum (Carr et al. 2018) (examples of studies in adolescents and adults: Blair et al. 2018; Boldrini et al. 2020; Coughlan et al. 2020; Korver‐Nieberg et al. 2013; Qiao et al. 2024, Bloomfield et al. 2021; studies by the Genetic Risk and Outcome of Psychosis Project—GROUP study such as de Haan and Schirmbeck 2023; Van Dam et al. 2014). An essential complementary approach is to assess attachment in early life, given that later‐life experiences could alter attachment patterns and the mental representations of the self and others (Bosmans et al. 2020). Attachment relationships established in infancy may affect representations of the self and others, and can have a significant impact throughout life (Berry et al. 2020). Studies have shown, for example, that experiences in childhood, including the relationship with the caregiver, can influence adult mental health and psychological functioning (Aafjes‐van Doorn et al. 2020; Massie and Szajnberg 2002), supporting a possible potential relation between variations in infant attachment and symptoms from the psychosis spectrum (Berry et al. 2020). Examining attachments in infancy and the quality of early caregiving can therefore provide evidence on how early‐life experiences may shape the neurodevelopmental and psychological origins of psychotic experiences (Berry et al. 2020). Thus, we contribute to addressing this gap in the literature by examining the prospective association between observed infant (disorganized) attachment and PEs in early (pre‐) adolescence.

Furthermore, a key predictor of infant attachment is parenting behavior (Ainsworth 1979; De Wolff and van IJzendoorn 1997; Madigan et al. 2024), and harsh parenting has also been related to offspring PEs (Fisher et al. 2012). In this study, we focused on two relevant parenting dimensions: (1) *extremely insensitive parenting*, which represents a threatening experience to the child and may constitute a relational trauma in early life (Mulder et al. 2017), and (2) *disconnected parenting*, in which parents show sudden interruptions of typical behavior by bouts of behavior disconnected from preceding behaviors and from context, which could be frightening to the child (Out et al. 2009). These parenting behaviors may cause fear and stress in the child and contribute to the development of disorganized attachment (Schuengel et al. 1999; van IJzendoorn et al. 1999) and, subsequently, of PEs (Radley et al. 2022).

An important antecedent of parenting, infant attachment, and PEs may be *parental loss experiences* (i.e., the parent losing an attachment figure, such as the partner or a child). Previous studies found some evidence that unresolved parental losses of an attachment figure may increase the likelihood of occurrence of parental frightened/frightening behavior (i.e., *disconnected parenting behavior*; Out et al. 2009; Schuengel et al. 1999), and this could, in turn, increase the risk for infant disorganized attachment (Main and Hesse 1990). In addition, parental loss experiences within 2 years around the child's birth have been related to offspring dissociative disorders in adulthood (Liotti 2004) as well as to offspring psychosis (Abel et al. 2014). Thus, we examined maternal loss experiences within the 2 years preceding or following the child's birth as the earliest exposure of interest, exploring whether maternal loss experiences were related to disconnected parenting, infant disorganized attachment, and subsequent child PEs.

In summary, using a sample of children from the general population, we examined the associations between maternal experiences of loss, disconnected and extremely insensitive parenting, and disorganized infant–parent attachment with the occurrence of PEs in childhood and adolescence. We hypothesized that maternal loss experiences, disconnected or extremely insensitive parenting, and infant disorganized attachment would be associated with a greater occurrence of PEs. We additionally hypothesized an indirect path linking maternal loss experiences with child PEs via parenting behaviors and disorganized attachment. We included two time‐point assessments of PEs to assess whether the relationship between our exposures of interest and the outcome differed across different developmental periods. However, considering that there might be more measurement error in the self‐report of PEs at age 10 years (Karcher 2022), and that hallucinations have the best predictive power for any PEs (Kelleher et al. 2009), we selected hallucinations at age 14 years as our main outcome of interest. Importantly, to maximize the generalizability and achieve a sample as large as possible, we assessed children from the general population. Previous studies found evidence for disconnected and extremely insensitive parenting occurring in low‐risk samples (Out et al. 2009) and parental unresolved losses in a considerable proportion of low‐risk participants (Bakkum et al. 2023). As our analyses provide evidence based on the general population, the conclusions would benefit from complementary research in high‐risk samples.

## 2 Materials and Methods

### 2.1 Participants

This study is embedded in the Generation R Study, a population‐based birth cohort that follows the development and health of children growing up in Rotterdam, the Netherlands (Kooijman et al. 2016). Pregnant mothers with a delivery date between April 2002 and January 2006 were invited to participate, and data were collected on multiple child and parent characteristics and general psychological and biological factors. Detailed data collection was performed in a subsample of children, the Generation R Focus subsample, whose parents and grandparents were all born in the Netherlands and whose mothers had a delivery date between February 2003 and August 2005 (*N* = 1232 as baseline) (Jaddoe et al. 2008). All participants provided written informed consent, and the study was approved by the Medical Ethics Committee of the Erasmus Medical Center, Rotterdam.

Among the Generation R Focus subsample, 882 children participated in the 14‐month visit, in which infant–parent attachment quality was observed. We excluded 24 children due to participation of siblings from the same family, and 29 children because of technical or procedural difficulties in the coding of attachment. Data on infant–parent attachment were available for 829 children, and from these, 627 children with available data on hallucinations at the 14‐year follow‐up (our main outcome of interest) were included in the analyses (Figure S1). Loss to follow‐up analysis is shown in Supporting Information.

### 2.2 Measures

#### 2.2.1 Attachment Quality

The quality of the infant–parent attachment relationship with the primary caregiver was assessed at 14 months of age with the validated and widely used Strange Situation Procedure (Ainsworth et al. 1978). This is based on the observation of infants during a mildly stressful task in which attachment behavior is triggered. The procedure includes eight 3‐min episodes, in which the parent leaves the infant in an unfamiliar room twice, first leaving the infant with a stranger, and second, leaving the infant alone. Because of logistical reasons, the pre‐separation episodes were slightly shortened without affecting the validity of the measures (Tharner et al. 2011). The infants’ behavior was rated by two trained coders, with inter‐rater agreement of 77% for the 4‐way attachment classification (kappa = 0.63) and of 87% for the disorganization coding (kappa = 0.64) (Tharner et al. 2011). The infants’ attachment pattern was classified into secure, insecure‐avoidant, insecure‐resistant, or insecure‐disorganized (Tharner et al. 2011), and a continuous score for disorganization was assigned (with higher scores indicating more attachment disorganization) (Main and Solomon 1990).

#### 2.2.2 Disconnected and Extremely Insensitive Parenting

These parenting behaviors were observed at the 14‐month research visit and assessed with the Disconnected and Extremely Insensitive Parenting (DIP) instrument (Out et al. 2009). Coders were unaware of the maternal sensitivity and attachment quality ratings. Parenting behaviors were assessed during a 12‐min psychophysiological evaluation and during the 5‐min lab visit break. In the psychophysiological evaluation, the child watched an episode of a children's TV program, while sitting on their caregiver´s lap and undergoing an ECG assessment. During the break, the caregiver and the child interacted freely (Mulder et al. 2017). Researchers observed whether the caregiver showed (1) disconnected behavior (i.e., unpredictable, sudden behavior disconnected from context and from previous behavior, including, for example, freezing, unexpected shifts in mood, and disoriented behaviors) and/or (2) extremely insensitive parenting (i.e., neglectful or aggressive behavior, that may be used as harsh discipline, such as lack of interaction with the child, lack of responsive behavior, or rough behaviors) based on the coding system by Out et al. (2009). The occurrence of each behavior was coded, and two final scores were assigned on a 9‐point scale, one for disconnected parenting and one for extremely insensitive parenting, with higher scores indicating more disconnected parenting and more extreme insensitivity, respectively. The intraclass correlation for intercoder reliability (single measure, absolute agreement) was 0.63 (*n* = 36) (Luijk et al. 2011).

#### 2.2.3 Maternal Experiences of Loss

Maternal experiences of loss within 2 years preceding or following the child's birth were assessed via multiple sources. First, mothers reported via questionnaires at 12–20 weeks of pregnancy whether they had ever had a miscarriage or a stillbirth and the year it occurred. Second, mothers reported via questionnaires at 20–25 weeks of pregnancy whether one of her children, her partner, her parents‐in‐law, her siblings, or one of her friends had passed away in the last 12 months. Third, mothers reported via questionnaire at child age 5 years whether they had ever had a miscarriage, a stillbirth, an abortion because of abnormality, or an abortion because of an unwanted pregnancy, and the year it occurred. Fourth, mothers reported in an interview at child age 10 years whether one of the child's caregivers had passed away and the year it occurred. This last measure was not included as no events occurred in our sample within the 2 years preceding or following the child's birth. Although miscarriage is a relatively common event (Quenby et al. 2021), evidence suggests that miscarriage may be associated with the presence of disorganized attachment behavior in children born after the loss, similar to the consequences of other parental losses of loved ones (Bakermans‐Kranenburg et al. 1999). We used two dichotomous variables to summarize maternal loss experiences within 2 years before or after the child's birth. One variable included all maternal loss experiences (i.e., fetal loss, loss of a child, a partner, a relative, or a friend; yes/no), and the other specifically focused on the loss of a close relative (i.e., fetal loss, loss of a parent, loss of a child; yes/no).

#### 2.2.4 Child Psychotic Experiences

##### 2.2.4.1 Hallucinations—Age 10 and 14 Years

The experience of hallucinations (*current* at age 10 years, and *during the preceding six months* at age 14 years) was self‐reported by the participant via two items from the Youth Self‐Report questionnaire: *“I hear sounds or voices that other people think aren't there”* and *“I see things that other people think aren't there”* (Achenbach 1991). The items could be rated as: *“not at all”* (0), *“a bit”* (1), or *“clearly”* (2). We used two dichotomous variables assessing the presence of any hallucinatory experiences (score > 0 on at least one item vs. score = 0 on both items) at age 10 years and at age 14 years (Bolhuis et al. 2023; Steenkamp et al. 2023). Our main outcome of interest was hallucinations at age 14 years.

##### 2.2.4.2 Delusions—Age 14 Years

The delusional experience was self‐reported via a questionnaire with six items from the Kiddie Schedule for Affective Disorder and Schizophrenia (Kaufman et al. 1997) (e.g., *“Have other people ever read your thoughts?”; “Have you ever believed that you were being sent special messages through television or radio?”*). Items could be rated as: *“No”* (0), *“Yes, probably”* (1), and *“Yes, certainly”* (2). We calculated a weighted total score if missingness in the items was below 25%, accounting for the number of completed items. The internal consistency was α = 0.58 (Steenkamp et al. 2023). Given the skewness of the sum scores, we dichotomized scores into delusional experiences present (score ≥ 3) or absent (score < 3) (Steenkamp et al. 2023). In total, 526 children out of the 627 (total sample) had data on delusions.

#### 2.2.5 Covariates

Based on the literature (e.g., Jones et al. 2018; Steenkamp, Tiemeier, Bolhuis, et al. 2021), we included the following covariates: child sex, child age at the PEs assessment, the highest education in the household, and maternal and paternal psychopathology at baseline. In addition, as in previous research (e.g., Steenkamp, Tiemeier, Bolhuis, et al. 2021), we accounted for whether the child received help (yes/no) in filling out the hallucinations questionnaire at age 10 years. Child sex and age were based on medical records. Maternal and paternal education were assessed in pregnancy via questionnaires and categorized based on the highest education in the household into low (primary school, secondary school, low or intermediate vocational training), middle (higher vocational training, bachelor's degree), and high (higher education). Maternal and paternal psychopathology were self‐reported at 20–25 weeks of pregnancy with the Brief Symptom Inventory (BSI) questionnaire (Derogatis 1993), a validated and widely‐used self‐reported inventory that addresses psychiatric symptoms occurring in the preceding 7 days. The BSI includes 53 items regarding psychiatric symptoms. The syndrome scales include somatization, obsession‐compulsion, interpersonal sensitivity, depression, anxiety, hostility, phobic anxiety, paranoid ideation, and psychoticism (Derogatis 1993). The maternal and paternal total sum scores, the Global Severity Index scores, were included in the analyses.

### 2.3 Statistical Analyses

**FIGURE 1:**
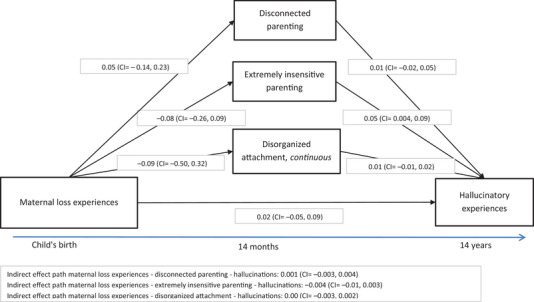
SEM model for the association between maternal loss experiences, parenting behaviors, disorganized attachment, and hallucinatory experiences. *Note*: *B* and 95% confidence intervals are presented. SEM model performed with estimator: MLR: robust maximum likelihood. In the lavaan package in R. Missing data accounted for with multiple imputation, and results pooled across 25 imputed datasets. Fit indices: robust CFI: 0.425, robust TLI: 1.000, robust RMSEA: 0.054. R‐squared: for hallucinations at 14 years: 0.04. *N* = 482. Paths adjusted for covariates: Path with disconnected parenting and the path with extremely insensitive parenting as the outcomes were adjusted for the highest household education + maternal psychopathology + paternal psychopathology at baseline. Path with disorganized attachment as the outcome adjusted for: child sex + highest household education + maternal psychopathology + paternal psychopathology at baseline. Path with hallucinations as the outcome adjusted for: child sex + age at the psychotic experiences assessment + highest household education + maternal psychopathology + paternal psychopathology at baseline.

First, we examined the Spearman correlations between all variables of interest. Second, we examined the association between attachment disorganization (dichotomous measure and continuous score) and PEs (hallucinations at age 10 and 14 years, and delusions at age 14 years) with logistic regression. The same approach was used for the other exposures (i.e., disconnected and extremely insensitive parenting, and maternal loss experiences). The analyses were performed in an unadjusted model and then in a model fully adjusted for covariates. Subsequently, we used structural equation modeling (SEM) to explore in one model the association between all variables of interest (maternal loss experiences, parenting behaviors, disorganized attachment, and hallucinations at age 14 years). We modeled the direct effect of maternal loss experiences on hallucinations at age 14 years, as well as the indirect path via disconnected and extremely insensitive parenting and disorganized attachment. The model is presented in Figure 1 and was examined in a model adjusted for covariates.

Model testing of the SEM was performed with the robust maximum likelihood estimator to account for potential non‐normality in our data. The hallucinations variable (coded 0/1) and all dichotomous covariates were coded as numeric in these analyses. The household's highest education variable was dummy‐coded. The SEM models were performed in the subset of children who participated with their mothers in the attachment and parenting behavior assessment, given that maternal loss experiences were hypothesized to affect the capacity of mothers to respond to the child in these two types of interactions (Out et al. 2009). To evaluate the robustness of our results, the SEM model was performed accounting for missing data based on multiple imputation (described below) and in a model using full information maximum likelihood (FIML). We used the *lavaan* package (Rosseel 2012) v.0.6‐19 and *semTools* v.0.5‐6.932 (Jorgensen et al. 2022).

The logistic regression analyses between disorganized attachment and hallucinations at age 14 years (our main outcome of interest) were adjusted in additional models for disconnected and extremely insensitive parenting to explore whether these parenting behaviors could explain any observed association. This adjustment was only exploratory and not included in the SEM model, given that parenting behaviors were assessed in the same research visit as attachment behaviors, therefore limiting the temporal interpretation of results.

All analyses were performed in R (R Core Team 2020) v.4.4.1. In the sample of children with complete data for attachment classification and hallucinations at 14 years (*n* = 627), missing data in covariates, exposures, and the other outcomes (hallucinations at 10 years and delusions) were accounted for with multiple imputation using the *mice* package (v.3.17.0) (van Buuren and Groothuis‐Oudshoorn 2011), and pooling results across 25 imputed datasets. The highest missingness was for the delusions measure (16.1%). The data for parenting behaviors were not imputed due to poor performance of the imputation models for these variables (see Methods in Supporting Information).

We conducted a sensitivity power analysis (using the R package WebPower v.0.9.4) to determine the minimum effect size that could be detected by our logistic regression models. We focused on infant disorganized attachment and hallucinations at age 14 years, as this was the main interest of the study. Based on our total sample of 627, an alpha of 0.05, and an outcome base rate of 0.11, we observed that our study had sufficient power (power = 0.85) to detect an odds ratio (OR) of 1.09 per each unit increase in disorganized attachment.

## 3 Results

Baseline characteristics are presented in Table 1. Overall, 51% of children had a secure attachment, 12% insecure‐avoidant attachment, 17% insecure‐resistant attachment, and 20% insecure‐disorganized attachment with their parent. Experiences of hallucinations were reported by 33% of children at age 10 years and by 12% of children at 14 years. Delusions were reported by 11% of children. Most of the children came from families with higher parental education (57%). In total, 24% of children had mothers who had experienced a loss within 2 years of the child's birth (72% of these cases were due to fetal loss), and 21% of children had mothers who had experienced a loss of a close relative in that period. Correlations between the main variables of interest are presented in Table S1.

**TABLE 1:** Sample characteristics.

	Mean (SD) or %
**Child characteristics**	
Sex, % girls	49.9
Disorganized attachment, median (IQR) score (observed range: 1–9)	3.5 (1, 4.5)
Attachment classification (%)	
Secure	50.9
Avoidant	12.4
Resistant	16.7
Disorganized	19.9
DIP, disconnected parenting, median (IQR)	1 (1, 1)
DIP, extreme insensitivity, median (IQR)	1 (1, 1)
Hallucinations at 10 years, % yes	33.1
Hallucinations at 14 years, % yes	11.6
Hallucinations assessment 10 years, % filled out alone	45.8
Delusions at 14 years, % yes	10.7
Age at hallucinations (10‐year assessment)	9.8 (0.3)
Age at hallucinations (14‐year assessment)	13.6 (0.4)
**Parental characteristics**	
Parent participating in attachment assessment	
Mother, %	86.0
Father, %	14.0
Highest education in the household, %	
Low	19.5
Medium	23.4
High	57.1
Maternal loss experiences, %	24.0
Maternal loss of a close relative, %	21.2
Maternal psychopathology, median (IQR)	0.12 (0.06, 0.21)
Paternal psychopathology, median (IQR)	0.06 (0.02, 0.14)

*Note*: Based on imputed data. Parenting behaviors (DIP) based on complete data. *N* = 627.

Abbreviation: IQR, interquartile range.

There was no evidence of an association between attachment disorganization and PEs (hallucinations at age 10 and 14 years, and delusions). Disorganized attachment, continuously scored, was not associated with hallucinations at 14 years (adjusted OR: 1.03, 95% CI 0.80, 1.33) (Table 2). Also, no association was observed between disconnected parenting and PEs, for example, children whose mothers showed more disconnected parenting did not have more hallucinations at 14 years (adjusted OR: 1.13, 95% CI 0.88, 1.44). In contrast, more extreme insensitive parenting was related to a greater risk of hallucinations at age 14 years (adjusted OR: 1.34, 95% CI 1.07, 1.66) and delusions (adjusted OR: 1.31, 95% CI 1.02, 1.68) (Table 3). Maternal loss experiences, assessed in the broader sense or focused on the loss of a close relative, were not related to any outcome (see Table 4). Also, there was no meaningful change in the association between disorganized attachment and hallucinations at age 14 years after adjustment for the parenting behaviors (Table S2).

**TABLE 2:** Association between attachment disorganization and child psychotic experiences.

Outcome (*N* cases)^a^	Exposure	Unadjusted model		Adjusted model^b^	
		Odds ratio (95% CI)	*p*	Odds ratio (95% CI)	*p*
Hallucinations at age 10 years, any (*N* = 207) vs. no	Disorganized attachment, continuous score	0.89 (0.75, 1.06)	0.18	0.91 (0.76, 1.09)	0.30
	Disorganized attachment, dichotomous score	0.75 (0.48, 1.18)	0.21	0.77 (0.49, 1.22)	0.27
Hallucinations at age 14 years, any (*N* = 73) vs. no	Disorganized attachment, continuous score	1.05 (0.82, 1.34)	0.68	1.03 (0.80, 1.33)	0.79
	Disorganized attachment, dichotomous score	1.25 (0.70, 2.25)	0.45	1.24 (0.68, 2.25)	0.48
Delusions at age 14 years, score of ≥ 3 (*N* = 67) vs. < 3	Disorganized attachment, continuous score	1.01 (0.77, 1.33)	0.95	1.00 (0.75, 1.32)	0.98
	Disorganized attachment, dichotomous score	0.83 (0.39, 1.73)	0.61	0.81 (0.38, 1.72)	0.58

*Note*: The continuous disorganized attachment is standardized. Disorganized attachment dichotomous score: 1 = disorganized, 0 = not disorganized. *N* = 627. In dichotomous disorganized attachment: yes = 125.

Abbreviation: CI, confidence intervals.

^a^ *N* pooled across imputed datasets.

^b^ Model adjusted for child sex + age at the psychotic experiences assessment + highest household education + maternal psychopathology + paternal psychopathology at baseline + help in filling out the questionnaire (only in models of hallucinations at age 10 years).

**TABLE 3:** Association between parenting behaviors and child psychotic experiences.

Outcome (*N* cases)	Exposure	Unadjusted model		Adjusted model^a^	
		Odds ratio (95% CI)	*p*	Odds ratio (95% CI)	*p*
Hallucinations at age 10 years, any (*N* = 164) vs. no	Disconnected parenting, continuous score	1.03 (0.84, 1.26)	0.77	1.04 (0.85, 1.28)	0.70
	Extreme insensitive parenting, continuous score	1.14 (0.94, 1.38)	0.19	1.19 (0.98, 1.46)	0.08
Hallucinations at age 14 years, any (*N* = 55) vs. no	Disconnected parenting, continuous score	1.11 (0.87, 1.41)	0.41	1.13 (0.88, 1.44)	0.34
	Extreme insensitive parenting, continuous score	1.32 (1.07, 1.63)	0.01	1.34 (1.07, 1.66)	0.01
Delusions at age 14 years, score of ≥ 3 (*N* = 48) vs. < 3	Disconnected parenting, continuous score	1.03 (0.75, 1.40)	0.87	1.00 (0.73, 1.37)	1.00
	Extreme insensitive parenting, continuous score	1.30 (1.02, 1.66)	0.03	1.31 (1.02, 1.68)	0.03

*Note*: The parental measures were standardized. *N* = 484. *N* pooled across imputed datasets.

Abbreviation: CI, confidence intervals.

^a^ Model adjusted for child sex + age at the psychotic experiences assessment + highest household education + maternal psychopathology + paternal psychopathology at baseline + help in filling out the questionnaire (only in models of hallucinations at age 10 years).

**TABLE 4:** Association between maternal loss experiences and child psychotic experiences.

Outcome (*N* cases)	Exposure	Unadjusted model		Adjusted model^a^	
		Odds ratio (95% CI)	*p*	Odds ratio (95% CI)	*p*
Hallucinations at age 10 years, any (*N* = 207) vs. no	Maternal loss experiences, dichotomous	1.11 (0.73, 1.70)	0.63	1.07 (0.70, 1.63)	0.76
	Maternal loss of a close relative, dichotomous	0.93 (0.59, 1.47)	0.76	0.89 (0.56, 1.41)	0.63
Hallucinations at age 14 years, any (*N* = 73) vs. no	Maternal loss experiences, dichotomous	1.20 (0.67, 2.16)	0.53	1.18 (0.65, 2.14)	0.58
	Maternal loss of a close relative, dichotomous	1.04 (0.55, 1.97)	0.90	1.04 (0.54, 1.98)	0.91
Delusions at age 14 years, score of ≥ 3 (*N* = 67) vs. < 3	Maternal loss experiences, dichotomous	1.15 (0.59, 2.23)	0.68	1.11 (0.56, 2.19)	0.77
	Maternal loss of a close relative, dichotomous	0.99 (0.48, 2.06)	0.99	0.94 (0.45, 1.97)	0.88

*Note: N* pooled across imputed datasets. Maternal loss experiences, yes = 150. Maternal loss of a close relative, yes = 133. Total *N* = 627.

Abbreviation: CI, confidence intervals.

^a^ Model adjusted for child sex + age at the psychotic experiences assessment + highest household education + maternal psychopathology + paternal psychopathology at baseline + help in filling out the questionnaire (only in models of hallucinations at age 10 years).

Figure 1 shows the SEM model including all variables of interest. The model showed a moderate‐to‐good fit to the data (robust CFI: 0.425, robust TLI: 1.000, robust RMSEA: 0.054). Of note, the relatively small sample size can alter fit indices even in correctly‐specified models (Shi et al. 2019). The results were consistent with those of the regression analyses, that is, the only significant association was between extremely insensitive parenting and more hallucinations at age 14 years (adjusted B: 0.05, 95% CI 0.004, 0.09). There was no direct path from maternal loss experiences to hallucinations at age 14 years, and also no indirect path via disconnected parenting: *B*_indirect effect_: 0.001 (95% CI −0.003, 0.004) or any other mediator. Similar estimates were obtained in the model using FIML to account for missing data and in the model with maternal loss of a close relative (Figures S2 and S3).

## 4 Discussion

This population‐based study of over 600 children offered a unique opportunity to prospectively evaluate the link between observed early parenting and infant–parent attachment with PEs in childhood and adolescence. We highlight three key results. First, contrary to our hypothesis, there was no evidence for an association between infant disorganized attachment and PEs in early adolescence. Second, exposure to more extreme insensitive parenting was consistently related to more hallucinations and delusions in adolescence, highlighting the importance of adverse caregiving experiences in the developmental pathway to PEs. Third, we found no evidence for the hypothesized pathway between maternal loss experiences around the time of childbirth, parenting behaviors and infant disorganized attachment, and the subsequent risk for child PEs.

Existing evidence supports a role of the environment in early life for the risk of subsequent psychopathology (Berg‐Nielsen et al. 2002; Straatmann et al. 2019), including PEs (Staines et al. 2022). For example, the experience of adversities during childhood, including physical and sexual abuse, and the exposure to harsh parenting have been found to be associated with a greater risk for PEs (Fisher et al. 2012; Sideli et al. 2020). However, most evidence for an impact of childhood adversity on PEs is based on parental reports on their own behavior, which may be affected by response biases (Grimm 2010; Runze and van IJzendoorn 2024). Moreover, although often hypothesized, the role of *early‐life* attachment in the development of psychosis has rarely been examined with a prospective design. Prospective studies have demonstrated a meta‐analytic association between disorganized attachment in infancy and an increase in externalizing behavioral problems in childhood (Groh, Fearon, et al. 2017). Here, we contribute to the evidence base for PEs with data based on *prospective* assessments with *observations* of parenting and infant attachment. Our results of extremely insensitive parenting are similar in effect direction to the previously reported association between child maltreatment and PEs (Fisher et al. 2012; Sideli et al. 2020), supporting the relevance of early adverse caregiving in relation to subsequent PEs in adolescence.

We found no evidence for disorganized attachment as an antecedent of PEs. We note that the confidence intervals were wide, as there were few children with PEs and high levels of disorganized attachment. However, our sensitivity power analysis suggests that we had sufficient power to detect an OR of 1.09, and thus the association of disorganized attachment and PEs in the general population might be absent or relatively small if detected in larger samples. Indeed, previous studies in population‐based cohorts have similarly found no evidence of a relation between early‐life relational problems between infants and their caregivers with PEs later in childhood (Clemmensen et al. 2016). Also, null results could be related to the lower stability of attachment from infancy to adolescence compared to parenting behavior (Beijersbergen et al. 2012).

Hesse and Main (2006) postulated that maternal traumatic experiences, such as maternal loss experiences, might lead to maternal frightened behavior which could result in infant disorganized attachment. In addition, maternal experiences of loss have been shown to predict a greater risk of psychosis in the offspring (Abel et al. 2014). However, we found no evidence in our sample for the pathway of maternal loss experiences leading to alterations in parenting or infant disorganized attachment, and lastly to child/adolescent PEs. We cannot rule out that this association is specific for certain subgroups. For example, a study showed that mothers with unresolved loss and an insecure representation of attachment displayed frightening maternal behavior, but not mothers with a secure attachment representation (Schuengel et al. 1999). However, these results were not consistently observed in a study using multiverse analyses (Schuengel et al., unpublished manuscript). Another possibility is that the association between maternal loss experiences and subsequent outcomes varies depending on the resolution of loss. We had no data on whether maternal losses were unresolved losses, and this may have diluted associations (Out et al. 2009). Also, when interpreting these findings, it is important to note that a large percentage of maternal loss experiences were related to fetal losses.

The importance of understanding the etiology of PEs lies in the relatively common prevalence of PEs in children and adolescents in the general population (Kelleher et al. 2012; Staines et al. 2022) and their link to a greater risk for subsequently developing psychotic disorders and other psychopathologies, substance abuse, and suicidal behavior (Healy et al. 2019; Staines et al. 2022). Our research on attachment and early parenting sheds light on potential antecedents of PEs, but also offers unique evidence on pathways that have been often hypothesized to explain the influence of childhood adversity on the risk for PEs (Sideli et al. 2020). The evidence supports a link between childhood adversities and PEs, but to date, studies evaluating the potential mediating role of attachment have mainly used cross‐sectional designs and focused on adult attachment style (e.g., Goodall et al. 2015; Pilton et al. 2016, GROUP study (Van Dam et al. 2014), and for a review see: Sideli et al. 2020), which corresponds to a different life period, other measures, and has temporal limitations. We recommend that to study the mediating role of attachment in the association of adversity and PEs, researchers assess attachment with observed measurements at various ages in childhood and use large sample sizes.

The strengths of this study are the observed infant–parent attachment and parenting behavior, and the prospective design of the study. However, there are some limitations that need to be considered. First, our sample had few cases with our exposures and some of the outcomes of interest, namely maternal loss experiences, severe alterations in parenting behavior, disorganized attachment, and PEs. This may have limited the statistical power to identify small associations. However, few severe cases are to be expected in samples from the general population. Also, this is the largest study to date on the topic and previous research in the Generation R cohort supports expected associations with other factors, for example, between disorganized attachment and cortisol levels (Luijk et al. 2010); between childhood adversities and PEs (Bolhuis, Koopman‐Verhoeff, et al. 2018). Second, our assessment of maternal loss experiences (2 years around birth) partially overlapped with the attachment assessment (assessed at 14 months). Also, we did not assess whether maternal loss experiences were *unresolved*, nor did the assessment of child PEs include information on the *distress* experienced. These two factors may be further examined in future studies. Research in larger samples could also explore the association between disorganized infant–parent attachment and persistent PEs (Steenkamp, Tiemeier, Blanken, et al. 2021). Third, we had no data on paternal loss experiences. Fourth, the generalizability to other populations needs to be examined. Fifth, PEs were self‐reported through questionnaires, which may have biased our PEs assessment. However, previous results based on this cohort support the validity of the instruments (e.g., Bolhuis, Kushner, et al. 2018; Steenkamp, Bolhuis, Blanken, et al. 2021), and research has shown that self‐report PEs questionnaires are a valid and reliable source of information in young adolescents, with clinical relevance and good predictive power for clinician‐rated PEs, specifically for items on hallucinatory experiences (Kelleher et al. 2009; Rimvall et al. 2019). Sixth, although we included several potential confounding factors, our results may still be affected by residual confounding from unmeasured or imperfectly measured variables, such as genetic factors, residual sociodemographic confounders, or underreported mental health issues.

## 5 Conclusion

We did not find support for the hypothesized role of infant–parent attachment quality in the development of PEs in adolescence. As we have argued elsewhere (van IJzendoorn and Bakermans‐Kranenburg 2024), observation of actual parent–child interactions might be a more powerful predictor of later development than the more volatile assessment of attachment in infancy. Importantly, our results provide supportive evidence for a role of early‐life extreme insensitive parenting in the origins of subsequent PEs.

## Author Contributions

A.P.C.H., M.J.B.‐K., and M.H.vIJ. formulated the research question. A.P.C.H., M.J.B.‐K., M.H.vIJ., K.B., and H.T. designed the study and interpreted the data. A.P.C.H. performed the data analyses, created the visualizations, and drafted the manuscript. All authors revised the manuscript, contributed to the interpretation of the results, and approved the manuscript for submission.

## Funding

Funding was provided by the Netherlands Organization for Scientific Research (M.H.vIJ.: NWO SPINOZA prize).

## Ethics Statement

All participants provided written informed consent, and the study was approved by the Medical Ethics Committee of the Erasmus Medical Center, Rotterdam.

## Conflicts of Interest

The authors declare no conflicts of interest.

## Supporting information




**Supplementary Materials**: brb371286‐sup‐0001‐SuppMat.docx

## Data Availability

The study has an open policy in regard to collaboration with other research groups. Requests for collaboration should primarily be pointed to Prof. Dr. Vincent Jaddoe (v.jaddoe@erasmusmc.nl). These requests are discussed in the Generation R Study Management Team regarding their study aims, overlap with ongoing studies, logistic consequences, and financial contributions. After approval of the project by the Generation R Study Management Team and the Medical Ethical Committee of the Erasmus Medical Center, the collaborative research project is embedded in one of the five research areas supervised by the specific principal investigator.
