# Supplementary material for: Early Parenting and Infant–Parent Attachment: Developmental Origins of Psychotic Experiences
Source: Brain Behav. 2026 Feb 28;16(3):e71286. doi: 10.1002/brb3.71286 (PMC12949724; doi:10.1002/brb3.71286)
Supplement: Supplementary file 1 — Supplementary Materials: brb371286‐sup‐0001‐SuppMat.docx [file BRB3-16-e71286-s001.docx]

**Supplementary Material for: Early Parenting and Infant-Parent Attachment: Developmental Origins of Psychotic Experiences**

**Supplementary Figure 1.** Flowchart of sample selection

Children with attachment classification available N = 829

**Children with information available on hallucinations at 14 years N= 627**

Children with no data on hallucinations at 14 years N = 202

**Children who participated with their mothers on the assessment of attachment and parenting behaviors N= 482**

**Supplementary Figure 2.** SEM model for the association between maternal loss experiences, parenting behaviours, disorganized attachment and hallucinatory experiences *(using FIML)*

Indirect effect path maternal loss experiences - disconnected parenting - hallucinations: 0.001 [CI= -0.002, 0.003]

Indirect effect path maternal loss experiences - extremely insensitive parenting - hallucinations: -0.005 [CI= -0.01, 0.003]

Indirect effect path maternal loss experiences - disorganized attachment - hallucinations: -0.001 [CI= -0.004, 0.002]

0.02 [CI= -0.05, 0.08]

Disconnected parenting

Extremely insensitive parenting

Maternal loss experiences

Disorganized attachment, *continuous*

Hallucinatory experiences

0.01 [CI= -0.01, 0.02]

-0.09 [CI= -0.27, 0.08]

0.05 [CI= -0.13, 0.22]

Child's birth

14 months

14 years

0.01 [CI= -0.02, 0.05]

0.05 [CI= 0.01, 0.09]

-0.11 [CI= -0.52, 0.30]

**Note.** B and 95% confidence intervals are presented. SEM model performed with estimator: MLR: Maximum Likelihood with robust standard errors and scaled test statistic. In Lavaan package in R. Missing values accounted for with full-information maximum likelihood. Fit indices: robust CFI: 0.488, robust TLI: -1.177, robust RMSEA: 0.051. R squared: for Hallucinations at 14y: 0.03. N = 482.

Paths adjusted for covariates:
Path with disconnected parenting and the path with extremely insensitive parenting as the outcomes were adjusted for highest household education + maternal psychopathology + paternal psychopathology at baseline.

Path with disorganized attachment as the outcome adjusted for: child sex + highest household education + maternal psychopathology + paternal psychopathology at baseline.

Path with hallucinations as the outcome adjusted for: child sex + age at the psychotic experiences assessment + highest household education + maternal psychopathology + paternal psychopathology at baseline.

**Supplementary Figure 3.** SEM model for the association between maternal loss experiences *(loss of a close relative)*, parenting behaviours, disorganized attachment and hallucinatory experiences *(using FIML)*

-0.01 [CI= -0.08, 0.05]

-0.08 [CI= -0.27, 0.11]

Disconnected parenting

Extremely insensitive parenting

Maternal loss of a close relative

Disorganized attachment, *continuous*

Hallucinatory experiences

Indirect effect path maternal loss of a close relative - disconnected parenting - hallucinations: 0.00 [CI= -0.002, 0.003]

Indirect effect path maternal loss of a close relative - extremely insensitive parenting - hallucinations: -0.004 [CI= -0.01, 0.004]

Indirect effect path maternal loss of a close relative - disorganized attachment - hallucinations: -0.001 [CI= -0.004, 0.002]

0.01 [CI= -0.02, 0.05]

0.01 [CI= -0.01, 0.02]

0.05 [CI= 0.01, 0.09]

Child's birth

14 months

14 years

-0.12 [CI= -0.57, 0.32]

0.02 [CI= -0.15, 0.20]

**Note.** B and 95% confidence intervals are presented. SEM model performed with estimator: MLR: Maximum Likelihood with robust standard errors and scaled test statistic. In Lavaan package in R. Missing values accounted for with full-information maximum likelihood. Fit indices: robust CFI: 0.477, robust TLI: -1.221, robust RMSEA: 0.051. R squared for Hallucinations at 14 years: 0.03. N = 482.

Paths adjusted for covariates:

Path with disconnected parenting and the path with extremely insensitive parenting as the outcomes were adjusted for highest household education + maternal psychopathology + paternal psychopathology at baseline.

Path with disorganized attachment as the outcome adjusted for: child sex + highest household education + maternal psychopathology + paternal psychopathology at baseline.

Path with hallucinations as the outcome adjusted for: child sex + age at the psychotic experiences assessment + highest household education + maternal psychopathology + paternal psychopathology at baseline.

**Supplementary Table 1.** Spearman correlations between the main variables of interest.

|  | **Hallucinations at 14 years** | **Hallucinations at 10 years** | **Delusions at 14 years** | **Disorganized attachment score** | **DIP, disconnected parenting** | **DIP, extreme insensitivity** | **Maternal loss experiences** | **Maternal loss of a close relative** | **Child sex** | **Age at hallucinations (10-year assessment)** | **Age at hallucinations (14-year assessment)** | **Highest household education** | **Maternal psychopathology** |
| --- | --- | --- | --- | --- | --- | --- | --- | --- | --- | --- | --- | --- | --- |
| **Hallucinations at 14 years** | - |  |  |  |  |  |  |  |  |  |  |  |  |
| **Hallucinations at 10 years** | 0.19*** | - |  |  |  |  |  |  |  |  |  |  |  |
| **Delusions at 14 years** | 0.25*** | 0.17*** | - |  |  |  |  |  |  |  |  |  |  |
| **Disorganized attachment score** | 0.04 | -0.05 | 0.01 | - |  |  |  |  |  |  |  |  |  |
| **DIP, disconnected parenting** | 0.09 | 0.05 | 0 | -0.13** | - |  |  |  |  |  |  |  |  |
| **DIP, extreme insensitivity** | 0.14** | 0.06 | 0.05 | -0.04 | 0.08 | - |  |  |  |  |  |  |  |
| **Maternal loss experiences** | 0.03 | 0.04 | 0 | -0.03 | 0.06 | -0.04 | - |  |  |  |  |  |  |
| **Maternal loss of a close relative** | -0.01 | -0.01 | -0.01 | -0.04 | 0.07 | -0.04 | 0.92*** | - |  |  |  |  |  |
| **Child sex** | -0.05 | -0.03 | 0.1* | 0.05 | 0.06 | 0.02 | 0.05 | 0.06 | - |  |  |  |  |
| **Age at hallucinations (10-year assessment)** | 0.02 | -0.05 | -0.02 | 0.08 | -0.04 | -0.07 | -0.08 | -0.08 | 0.1* | - |  |  |  |
| **Age at hallucinations (14-year assessment)** | -0.09 | -0.02 | 0 | 0.02 | 0 | -0.02 | -0.11* | -0.09 | -0.01 | 0.14** | - |  |  |
| **Highest household education** | -0.11* | 0.07 | -0.01 | -0.12** | 0.02 | -0.05 | -0.01 | 0.01 | 0.07 | 0.01 | 0.05 | - |  |
| **Maternal psychopathology** | -0.01 | 0.07 | 0.08 | 0.02 | -0.02 | -0.13** | 0.08 | 0.07 | -0.03 | -0.02 | 0.01 | -0.13** | - |
| **Paternal psychopathology** | 0.05 | 0.11* | 0.01 | -0.02 | 0.03 | -0.02 | 0.04 | 0.01 | 0.01 | 0.1* | 0.01 | -0.09 | 0.17*** |

Sex: boy:0, girl: 1; Maternal loss experiences: no: 0, yes: 1, Household education: low: 0, middle:1, high: 2. Hallucinations: absent:0, present:1, delusions: absent:0, present:1. Based on the first imputed dataset. N = 482. *: P-value <0.05, **: P-value <0.01, ***: P-value <0.001

**Supplementary Table 2.** Adjustment for parenting behaviors in the association between attachment disorganization and child hallucination experiences at age 14 years.

| **Outcome (N cases)*** | **Exposure** | **Main model**** | | **Additionally adjusted models***** | | | |  |
| --- | --- | --- | --- | --- | --- | --- | --- | --- |
|  |  |  |  | **Adjusted for disconnected parental behavior** | | **Adjusted for extreme insensitive parenting** | |  |
|  |  | **Odds Ratio (95% CI)** | **p** | **Odds Ratio (95% CI)** | **p** | **Odds Ratio (95% CI)** | **p** |  |
| Hallucinations at age 14 years, any (N = 55) vs no | Disorganized attachment, continuous score | 1.08 (0.81; 1.44) | 0.58 | 1.10 (0.83; 1.47) | 0.50 | 1.11 (0.83; 1.48) | 0.49 |  |
| The continuous disorganized attachment score is standardized. N = 484. *N pooled across imputed datasets. CI = confidence intervals | | | | | | | |  |
| ** Model adjusted for child sex + age at the psychotic experiences assessment + highest household education + maternal psychopathology + paternal psychopathology at baseline ***Main model additionally adjusted for: 1. disconnected parental behavior, 2. extreme insensitive parenting | | | | | | | |  |
|  |  |  |  |  |  |  |  |  |

**Supplementary Methods**

**Statistical analyses**

The parenting variables showed insufficient convergence during the multiple imputations, based on the distribution of the imputed values. The diagnostic trace plots for the mean and standard deviation of the imputed parenting variables showed definite trends, and the kernel density estimates of the imputed vs observed data for this variable showed distributions that did not correspond correctly (van Buuren, 2018). Given the lack of predicting variables that could improve the imputation, missing data for the parenting variables were not imputed.

**Supplementary Results**

**Lost-to-follow-up analysis**

We compared children in the study sample (N = 627) to children who had infant attachment data available, but no data on hallucinations at age 14 years (our main outcome of interest, N = 202). We used chi-squared tests for categorical variables and Mann-Whitney test for continuous variables. Children included in analyses did not differ from those with no hallucinations data in child sex (girls: study sample: 49.9%, lost-to-follow-up sample: 47.5%, p = 0.61), or in the disorganized attachment score (median (interquartile range): study sample: 3.5 (1.0, 4.5), lost-to-follow-up sample: 3.5 (1.0, 5.0), p = 0.08). The distribution of the highest household education did not differ between groups (p = 0.09).
